# Health Control Beliefs and Attitude Toward Treatment in Psychiatric and Non-Psychiatric Clinical Samples

**DOI:** 10.3389/fpsyt.2021.537309

**Published:** 2021-04-29

**Authors:** Laszlo Pogany, Judit Lazary

**Affiliations:** ^1^National Institute of Mental Health, Neurology and Neurosurgery, Budapest, Hungary; ^2^Janos Szentagothai Doctoral School of Neuroscience, Budapest, Hungary

**Keywords:** compliance, adherence, drug attitude, health belief, psychiatric treatment

## Abstract

Although there is accumulating evidence on the potential influencing factors of medication adherence, the knowledge about patients' attitudes and beliefs toward treatment is only partly utilized in adherence-improving strategies. Several internal and external factors determining adherence have been described regarding many chronic somatic diseases but in recent research, insight on psychiatric patients has been exclusively lacking. As a result, there is a scarcity of effective adherence-improving interventions. Identification of any specific differences or similarities between the attitudes toward treatment of psychiatric and non-psychiatric patients would help to support adherent behavior.

We recruited 189 participants from four departments of general psychiatry (GEN PSYCH, *n* = 106), addictology (ADDICT, *n* = 42) and somatic diseases (NON PSYCH, *n* = 41). The Patient's Health Belief Questionnaire on Psychiatric Treatment (PHBQPT) was performed to assess the patients' attitude toward drug treatment, perceived health locus of control, and psychological reactance.

The most robust difference of the PHBQT scores occurred between the GEN PSYCH and ADDICT subgroups. ADDICT patients scored significantly higher on the internal and external health locus of control and on the Psychological Reactance subscale as well. While GEN PSYCH subjects provided higher scores on the Positive Aspect of Medication compared to ADDICT persons. Interestingly, the only difference between the GEN PSYCH and NON-PSYCH groups was the more pronounced mistrust in physicians in the case of psychiatric patients.

Our data suggest that mistrust toward medication does not differ in psychiatric and non-psychiatric samples, while the acceptance of the doctor's competency may be stronger in the non-psychiatric sample. The analysis of these factors provides information which could help us better understand this important issue and to develop more efficient interventions for improving adherence.

## Introduction

Medication non-adherence is one of the most challenging problems of healthcare. In the case of patients requiring psychiatric treatment, non-adherence is considered to be one of the major factors which influence the course of illness and the outcome. According to a report from the WHO, about 50% of patients with a chronic disease are non-adherent to their medication (1) and some authors suggest that improving adherence at a population level may have a higher influence on health than the discovery of a new compound (2). Detailed reviews are available about the factors influencing adherence (3–8) and there are some interesting data on the mediating role of depression on adherence in chronic somatic diseases, such as hypertension (9), cardiovascular disease (2), diabetes (10), and chronic kidney disease (11). However, few direct comparisons have been made between somatic and psychiatric patients' adherence. Further evidence suggested that health-related locus of control can moderate the relationship between a serious somatic disease causing disability and the consecutive occuring depressive symptoms. Authors emphasized the importance of assessment of control beliefs of patients undergoing treatment for serious somatic illness in order to facilitate the recovery process (12).

Medication taking is a complex behavior and several potentially influencing factors have been investigated and proven to correlate with it. The results have shown that demographic, medical, or personality factors have less influence on adherence than psychological and emotional aspects. Comprehensive analysis of these factors led to the recognition of the importance of beliefs about medication's impact on health (13). Further studies confirmed the crucial role of social cognition in medication adherence and Social Cognition Models appeared in the literature of medication adherence (14). One of these concepts is the Health Locus of Control (HLOC) which has been identified as a determining dimension of medication adherence in several fields of medicine (4, 15). The Multidimensional Health Locus of Control Scale was developed by Wallston, Wallston, and de Vellis in order to measure HLOC. HLOC refers to “the degree to which individuals believe that their health is controlled by internal vs. external factors” Wallston et al. (25). The external factor has been divided into two subfactors, such as “chance or fate” and “powerful others.” Drug attitude and health control belief have been intensively studied in numerous fields of medicine in order to understand its complexity and impact on adherence. Self-efficacy also plays a positive role in developing patients' attitudes toward treatment. Patients with higher beliefs in their capabilities (higher self-efficacy) are more likely to be pharmacophilic (16).

Medication adherence can be negatively influenced by side effects, stigma, fear of addiction, previous negative experiences, negative attitudes toward drug treatment, and poor insight (17–19). In an exploratory study of adherence of schizophrenic patients it was found that the patients believing that their illness can be controlled by themselves and/or by their physicians were more likely to follow their prescriptions. The link between health locus of control and adherence appeared to be refined by insight (20). According to the results of one study, psychiatric patients' attitudes toward medication could be negatively influenced by educational level, patients with a higher educational level have been shown to be more skeptical about the usefulness of psychoactive drugs (16).

In this pilot study we investigated the role of beliefs and health locus of control in influencing the attitude toward pharmacological treatment in samples of patients with general psychiatric, addictive, and somatic disorders.

## Methods

### Study Sample

A total of 195 participants were recruited from four different departments of general psychiatry (Departments A, B, C, and D; hereinafter together referred to as GEN PSYCH, *n* = 112), a department of addictology (ADDICT, *n* = 42), a department of internal medicine (IM, *n* = 20), and a department of neurology (NEUR, *n* = 21) of the Nyíro Gyula National Institute of Psychiatry and Addictions, Budapest, Hungary. A dataset of 189 subjects were entered into the final statistical analysis, as six patients were excluded because of missing data (106 from GEN PSY + 42 from ADDICT + 41 subjects from IM+NEUR depts). Subjects were asked to participate in the survey anonymously. Patients treated at the department of addictology were admitted to the department after a motivational interview. Participants from the GEN PSYCH were diagnosed with psychiatric disorders coded ICD F2, F3, and F4. All patients were voluntarily treated. Patients being treated with at least one psychotropic medication (generally an anxiolytic) from IM and NEUR were selected to participate in the study. For the comparative analysis of different clinical samples with altered adherence characteristics, three subgroups were created: patients from the departments of general psychiatry (GEN PSYCH); department of addictology (ADDICT); and patients from the departments of internal medicine and neurology (NON-PSYCH). The study was approved by the Hungarian Central Ethical Committee, Budapest, Hungary (number of approval: 45735-5/2020). A signed informed consent was obtained from all subjects. The study was conducted according to the Declaration of Helsinki.

### Questionnaires

We used the Hungarian version of the Patient's Health Belief Questionnaire on Psychiatric Treatment (PHBQPT) to assess the patients' attitude toward drug treatment, perceived health locus of control, and psychological reactance developed by Armitage and Conner (14), De las Cuevas et al. (21), Poganyet al. (22). The questionnaire contains 17 items from three previously validated scales, the Drug Attitude Inventory (DAI-10) (23), the Hong Psychological Reactance Scale (HPRS) (24), and the Multidimensional Health Locus of Control (MHLC, Form C) (25) on five subscales (Positive Aspects of Medications, Negative Aspects of Medications, Doctor-HLOC, Internal-HLOC, and Psychological Reactance). Patients can rate on a 6-point Likert scale the degree to which they agreed or disagreed with the statements, from strongly disagree to strongly agree. The Hungarian version of the PHBQPT questionnaire was validated by our research group.

Originally, this newly developed questionnaire was validated in a sample of psychiatric patients. In the present study we decided to use this instrument also in the case of patients treated at internal medicine and neurology departments who were taking at least one psychotropic agent. The patients involved in the study were hospitalized at one of the four departments of the general psychiatry, were treated for addictions, or underwent treatment at the internal medicine and neurology department. The involvement of these different subpopulations aimed to compare the potential effects of the awareness of being treated for a psychiatric illness on the beliefs and attitude toward medication treatment.

### Data Analysis

To test the distribution of the scores we used the Kolmogorov-Smirnov test. To assess the differences between clinical subgroups (GEN PSYCH vs. ADDICT and GEN PSYCH vs. NON-PSYCH), the mean scores of items and subscales of the PBHQPT were compared using one-way ANOVA or *t*-test in the case of normal distribution. For comparing the mean scores between two groups we used the Mann-Whitney U test. The ANOVA test and *post hoc* Tukey's test was performed to analyse the differences among the mean scores of the three groups.

Chi-square tests were performed in the case of binary categorical variables. The effects of age and gender on the dependent variables were calculated by a general linear model. Missing data were excluded from the statistical analyses. Results were accepted as significant if the α-level was <0.05. All statistic tests were run in the SPSS 24.0 program.

## Results

### Descriptive Statistics

The distribution of mean scores deviated significantly from normal in the case of all subscales of PHBQPT according to the Kolmogorov-Smirnov test (*p* > 0.001 in all cases). The mean age of the NON-PSYCH subsample was significantly higher than of the GEN PSYCH or ADDICT subpopulations (*p* < 0.05). Gender ratio was equilibrated in the PSYCH subsample, but women were overrepresented in the NON-PSYCH sample (*p* = 0.012). The scores on the PHBQPT items and subscales showed similar trends with the results of De las Cuevas et al. (21). The effect of age and gender were tested by general linear models on the PHBQPT subscales. We found that in the NON-PSYCH sample only Psychological Reactance depended on the gender (*p* = 0.045). However, age had a significant effect on the Doctor-HLOC subscale in both PSYCH subsamples (*p* = 0.025; *p* = 0.023; respectively). Interestingly, Internal-HLOC was gender-dependent only in the ADDICT subgroup (*p* = 0.026). The detailed results of descriptive statistical analyses are shown in Tables 1, 2.

**Table 1:** Mean age and gender prevalences in the investigated subsamples.

	***N***	**Age (mean ± S.D.)**	**Gender (male/female)**
PSYCH (1)	148	46.7 ± 14.7	74/74
GEN PSYCH (2)	106	48.0 ± 15.6	48/58
ADDICT (3)	42	43.2 ± 11.8	28/14
NON-PSYCH (4)	41	66.8 ± 13.5	11/30
*p*-value		(1) vs. (4) <0.001	(1) vs. (4) 0.012

*Differences between groups were calculated by t-test and chi square tests*.

**Table 2:** Effect of gender and age on the PHBQPT subscales in the GEN PSYCH, ADDICT, and NON-PSYCH subsamples.

	**TOTAL SAMPLE**		**GEN PSYCH**		**ADDICT**		**NON-PSYCH**	
	**Gender**	**Age**	**Gender**	**Age**	**Gender**	**Age**	**Gender**	**Age**
Positive Aspects of Medication	NS	NS	NS	NS	NS	NS	NS	NS
Negative Aspects of Medication	NS	NS	NS	NS	NS	NS	NS	NS
Doctor-HLOC	NS	0.014	NS	0.025	NS	0.023	NS	NS
Internal-HLOC	0.027	NS	NS	NS	0.026	NS	NS	NS
Psychological Reactance	NS	NS	NS	NS	NS	NS	0.045	NS

*Effect of age and gender on PHBQPT subscale variances were tested by regression models*.

*PHBQPT, Patient's Health Belief Questionnaire on Psychiatric Treatment*.

**Figure 1:**
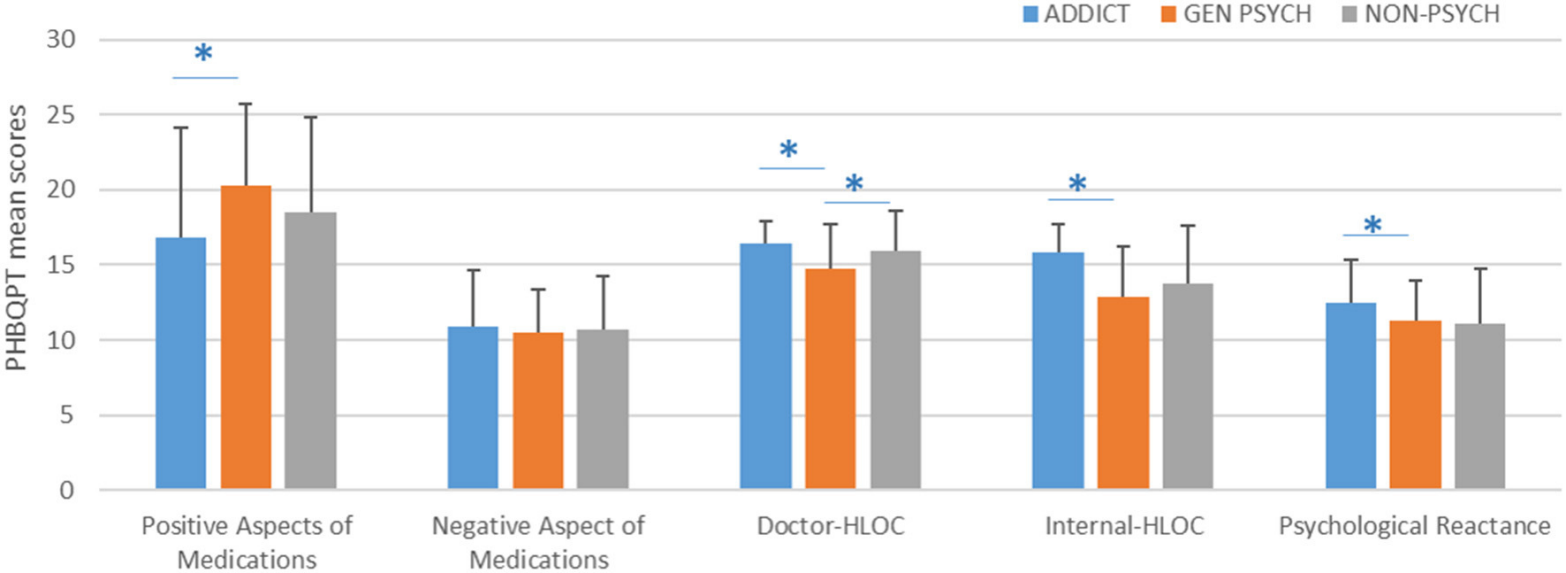
PHBQPT subscale scores in different clinical subgroups. ^*^p > 0.05 according to *post hoc* Tukey's test. Error bars represent the standard deviations (S.D.) of means.

Regarding the associations of mean scores of the PHBQPT subscales among the three clinical samples, we found significant associations in the case of Positive Aspect (*p*_ANOVA_ = 0.004), Doctor HLOC (*p*_ANOVA_ = 0.002); Internal HLOC (*p*_ANOVA_ = 8x10^−7^), and Psychological Reactance (*p*_ANOVA_ = 0.43) (Figure 1). Results of the *post hoc* paired-samples tests are described in sections Comparison of the PHBQPT scores in the GEN PSYCH and ADDICT clinical subsamples and Comparison of the PHBQPT scores in the GEN PSYCH and NON-PSYCH clinical subsamples.

### Comparison of the PHBQPT Scores in the GEN PSYCH and ADDICT Clinical Subsamples

Regarding pronounced differences in the attitude toward medication in patients with addictions, first we compared the PBHQPT scores between GEN PSYCH and ADDICT subgroups. As it was expected, participants treated for an addiction gave significantly different responses on almost all items of the questionnaire. Patients with addictions scored significantly higher on items 1, 2, 3, 6, 7, 8, 12, and 14 (all *p*-values < 0.05). In contrast, robustly higher scores were given by GEN PSYCH subgroup members on items 4, 11, 15, and 16 (all *p*-values < 0.05). Significantly higher scores on the Positive Aspects of the Medications subscale were found in the GEN PSYCH subpopulation while there was no difference in Negative Aspect between the two subsamples. However, patients with addictions scored significantly higher on the Doctor-HLOC, Internal-HLOC, and Psychological Reactance subscales (all *p-*values < 0.05). Results of the comparative analyses between GEN PSYCH and ADDICT subsamples are presented in Table 3 and Figure 1.

**Table 3:** Comparison of PHBQPT item scores and subscales between ADDICT vs. GEN PSYCH subsamples.

	**GEN PSYCH**	**ADDICT**	***p*-value**
1. I am directly responsible for my condition getting better or worse	4.8 ± 1.3	5.5 ± 0.8	0.001
2. If I see my doctor regularly, I am less likely to have a problem with my condition	4.6 ± 1.4	5.2 ± 1.3	0.007
3. When someone forces me to do something, I feel like doing the opposite	2.9 ± 1.4	3.7 ± 1.4	0.004
4. For me, the good things about medication outweigh the bad	4.3 ± 1.3	3.4 ± 1.8	0.001
5. I feel strange, “doped up”, on medication	3.7 ± 1.3	3.5 ± 1.8	NS
6. The main thing which affects my condition is what I myself do	4.3 ± 1.3	5.3 ± 0.8	0.000
7. Following doctor's orders to the letter is the best way to keep my condition from getting any worse	5.1 ± 1.1	5.6 ± 0.5	0.003
8. I resist the attempts of others to influence me	4.3 ± 1.4	4.8 ± 1.4	0.041
9. Medications make me feel more relaxed	4.2 ± 1.3	3.8 ± 1.7	NS
10. Medications make me feel tired and sluggish	3.6 ± 1.7	3.6 ± 1.6	NS
11. I feel more normal on medication	4.2 ± 1.4	3.5 ± 1.7	0.011
12. If my condition takes a turn for the worse, it is because I have not been taking proper care of myself	3.9 ± 1.5	4.9 ± 1.2	0.000
13. Whenever my condition worsens, I should consult a medically trained professional	5.3 ± 1.1	5.6 ± 0.7	NS
14. It is unnatural for my mind and body to be controlled by medication	3.4 ± 1.6	4.1 ± 1.9	0.012
15. My thoughts are clearer on medication	3.6 ± 1.6	2.7 ± 1.7	0.002
16. Taking medication will prevent me from having a breakdown	4.0 ± 1.4	3.3 ± 1.7	0.015
17. I become angry when my freedom of choice is restricted	4.2 ± 1.3	4.0 ± 1.6	NS
**Subscales**			
*Attitudes toward medication*			
Positive Aspects of Medication	20.3 ± 5.4	16.8 ± 7.3	0.001
Negative Aspects of Medication	10.5 ± 2.9	10.9 ± 3.8	NS
*Perception of health controls*			
Doctor-HLOC	14.8 ± 2.9	16.4 ± 1.5	0.001
Internal-HLOC	12.9 ± 3.3	15.8 ± 1.9	0.000
Psychological Reactance	11.3 ± 2.7	12.5 ± 2.8	0.012

*Mean scores were compared with Mann-Whitney U tests*.

*PHBQPT, Patient's Health Belief Questionnaire on Psychiatric Treatment*.

### Comparison of the PHBQPT Scores in the GEN PSYCH and NON-PSYCH Clinical Subsamples

Considering the major differences between the GEN PSYCH and ADDICT subsamples, we performed comparison analysis of the PHBQPT scale in the NON-PSYCH sample only vs. the GEN PSYCH subgroup. In this analysis the highest degree of agreement was found for the item “Whenever my condition worsens, I should consult a medically trained professional” in both subsamples (5.3 ± 1.1 and 5.6 ± 0.7; *p* > 0.05), similar to the results of the study of De las Cuevas et al. (21). However, psychiatric patients considered it significantly less important to follow their physician's suggestions (5.1 ± 1.0 vs. 5.5 ± 0.8; *p* = 0.03) and found regular visits to their doctors to be less effective (4.6 ± 1.4 vs. 5.2 ± 1.1; *p* = 0.010). Surprisingly, resistance against others' influence was more pronounced among patients with somatic disorders than in the PSYCH subgroup (4.3 ± 1.4 vs. 5.0 ± 1.6; *p* = 0.01). Concerning the PHBQPT subscales, NON-PSYCH participants scored signficantly higher on the Doctor-HLOC subscale compared to the GEN PSYCH group (15.3 ± 2.7 vs. 15.9 ± 2.7; *p* = 0.04). The results of comparative analysis between the GEN PSYCH and NON-PSYCH samples are presented in Table 4 and Figure 1.

**Table 4:** Comparative analysis of the PHBQPT items and subscales between the GEN PSYCH and NON-PSYCH subsamples and results of De las Cuevas et al. (21).

	**GEN PSYCH**	**NON-PSYCH**	***p*-value**	**De las Cuevas**
1. I am directly responsible for my condition getting better or worse	4.8 ± 1.3	4.9 ± 1.4	NS	4.7 ± 1.7
2. If I see my doctor regularly, I am less likely to have a problem with my condition	4.6 ± 1.4	5.2 ± 1.1	0.010	4.6 ± 1.7
3. When someone forces me to do something, I feel like doing the opposite	3.0 ± 1.4	2.9 ± 1.8	NS	2.7 ± 1.8
4. For me, the good things about medication outweigh the bad	4.4 ± 1.6	4.8 ± 1.1	NS	4.6 ± 1.7
5. I feel strange, “doped up”, on medication	3.7 ± 1.5	3.8 ± 1.8	NS	3.1 ± 2.0
6. The main thing which affects my condition is what I myself do	4.4 ± 1.3	4.7 ± 1.7	NS	4.2 ± 1.8
7. Following doctor's orders to the letter is the best way to keep my condition from getting any worse	5.1 ± 1.0	5.5 ± 0.8	0.03	4.9 ± 1.5
8. I resist the attempts of others to influence me	4.3 ± 1.4	5.0 ± 1.6	0.01	3.3 ± 1.9
9. Medications make me feel more relaxed	4.1 ± 1.4	3.8 ± 1.7	NS	4.9 ± 1.5
10. Medications make me feel tired and sluggish	3.6 ± 1.5	3.6 ± 1.7	NS	3.7 ± 2.0
11. I feel more normal on medication	4.0 ± 1.5	4.4 ± 1.4	NS	4.3 ± 1.8
12. If my condition takes a turn for the worse, it is because I have not been taking proper care of myself	3.9 ± 1.5	4.2 ± 1.7	NS	4.2 ± 1.9
13. Whenever my condition worsens, I should consult a medically trained professional	5.3 ± 1.1	5.6 ± 0.7	NS	5.5 ± 1.2
14. It is unnatural for my mind and body to be controlled by medications	3.6 ± 1.7	3.9 ± 1.8	NS	2.9 ± 1.9
15. My thoughts are clearer on medication	3.4 ± 1.6	3.1 ± 1.7	NS	4.0 ± 1.9
16. Taking medication will prevent me from having a breakdown	3.8 ± 1.5	2.9 ± 1.8	NS	4.1 ± 1.9
17. I become angry when my freedom of choice is restricted	4.2 ± 1.3	3.9 ± 1.8	0.002	4.2 ± 1.8
**Subscales**				
*Attitudes toward medication*				
Positive Aspects of Medication	19.3 ± 6.2	18.5 ± 6.3	NS	18.1 ± 4.8
Negative Aspects of Medication	10.6 ± 3.1	10.7 ± 3.6	NS	9.7 ± 4.2
*Perception of health control*				
Doctor-HLOC	15.3 ± 2.7	15.9 ± 2.7	0.04	15.1 ± 3.4
Internal-HLOC	13.8 ± 3.2	13.8 ± 3.8	NS	12.9 ± 4.2
Psychological Reactance	11.6 ± 2.7	11.1 ± 3.7	NS	10.2 ± 3.8

*Significance of differences of items and subscale scores were tested by Mann-Whitney U tests*.

*PHBQPT, Patient's Health Belief Questionnaire on Psychiatric Treatment*.

## Discussion

This is the first report on a comparative analysis of the drug attitude and health concept of different clinical samples. Despite the fact that drug adherence is a hot topic in clinical psychopharmacology and it is generally considered that psychiatric patients are less adherent with their treatment and the mistrust toward medication is more common among them compared to the non-psychiatric patient population, there have not been any direct comparisons regarding these aspects in psychiatric and non-psychiatric subsamples so far. The analysis of different samples may provide valuable information which could help us better understand the specific features and the general common mechanisms behind the attitudes toward treatment of patients suffering from different chronic diseases. This information can be used to develop more efficient interventions for improving adherence.

Poor treatment adherence leads to an enormous healthcare and economical burden. According to Krueger et al. (26), self-reported data overestimate medication adherence in clinical practice by as much as 200%. Lapane et al. (27) demonstrated that while doctors estimated that 9% of patients do not talk about their non-adherence, in reality 83% of patients reported that they would never tell their physician if they did not plan on picking up a prescription. According to some financial analyses, non-adherence leads to a loss of 100–300 billion dollars in the United States annually (IMS Institute for Healthcare Informatics). Certain estimations suggest that improving adherence to diabetes medication would prevent 699,000 emergency department visits and 341,000 hospitalizations each year in the United States of America (28). Some statistical data suggest that 33–69% of the hospitalizations are related to poor adherence (29).

The results of our study have shown that there is a more robust difference regarding attitude toward medication between ADDICT patients and GEN PSYCH patients than between the latter population and NON PSYCH subjects. Although positive aspects of medication appeared more pronounced in the GEN PSYCH sample, the trust in doctors and feelings of personal responsibility for their own health were presented at a higher level among patients with addictions. Participants belonging to the GEN PSYCH sample were more skeptical regarding the importance of seeing their physician than patients treated due to somatic diseases, as it is shown by the reduced Doctor-HLOC subscale score. GEN PSYCH patients do not believe that regularly seeing their doctor would decrease the risk of getting worse and they do not think that “following the doctor's order to the letter” is the best way to keep their condition from getting any worse. Analyzed together with other items of the scale, it can be concluded that GEN PSYCH patients believe that they might need some help, but they frequently refuse to follow the instructions of the medical professionals. However, according to the Positive and Negative Aspect subscale scores, beliefs and attitude toward medication do not differ significantly in the GEN PSYCH and NON-PSYCH samples.

In conclusion, the attitude toward medication was more positive in the GEN PSYCH than in the ADDICT subsamples, and similar in the GEN PSYCH and NON-PSYCH samples. Concerning the health control locus, the strongest external and internal health control belief appeared in the ADDICT subgroup while only the Doctor HLOC was higher in the NON-PSYCH than in the GEN PSYCH sample. Nevertheless, attitude toward medication is partially associated with beliefs and health locus of control.

Despite the fact that in the NON-PSYCH sample there were higher levels of external locus of control than in the psychiatric group, it seems that the level of mistrust toward medication is similar in both samples. These results are in concordance with the conclusions of a review published by Brown et al. (5). They emphasized that besides individual experiences the increasing mistrust of societies toward healthcare systems contribute to the negative beliefs. The authors highlighted that patients' negative beliefs are often stronger than their clinicians would suppose. An important factor contributing to patients' mistrust is the assumed relationship between the pharmaceutical companies and doctors. Grande et al. reported that 55% of patients believed that their doctors received gifts from the companies and this belief was associated with lower trust in their physician and doubled the chance of mistrust in the entire healthcare system (30). Another factor leading to mistrust was the contradiction between the information acquired from different sources (healthcare provider, media, internet) (5). De las Cuevas et al. (31) found that highly psychological reactant patients were more likely to be noncompliant; they generally resist any guidance or assistance.

Mago et al. (32) reported that in a sample of 2,096 subjects suffering from major depressive disorder, the most frequent negative emotion reported by patients regarding their medication was frustration (29.8% of respondents). Concerning feelings about their healthcare providers, the majority reported feeling understood, trust, and confidence but almost 20% reported frustration due to not feeling heard, ineffective treatment, and feeling rushed/lack of quality visit. The reasons for frustration with medication were lack of effectivity and tolerability issues. In contrast, physicians estimated that only 11% of patients were frustrated with their medications and 5% with the healthcare quality (32). In another survey, data of 3,684 subjects were analyzed concerning compliance to antidepressant treatment. They found that 22% was the overall level of compliance, thus only one in four patients complied with treatment. Surprisingly, better compliance was observed in patients with polypharmacy (33).

Understanding patients' health beliefs and attitudes toward drug treatment is needed for successful therapy. It is also essential that patients who are in their first contact with mental health care professionals are given comprehensive and appropriate information regarding the planned treatment (34).

Nevertheless, there are some limitations in this study, as, in the case of the patients recruited from the somatic departments, the sample size was smaller since the mean age of patients treated at the internal medicine and neurological departments was higher and their general health status was worse. The occurrence of neurocognitive disorders was higher in this group compared to the group of patients treated at departments of general psychiatry. Thus, we found fewer patients who were eligible for enrollment into the study. Another limitation is that we did not investigate treatment adherence (all patients were hospitalized throughout the entire duration of the study). On the other hand, it is the strength of our study that we are among the first researchers to have used the recently developed PHBQPT questionnaire in different clinical samples.

In conclusion, mistrust toward medication did not differ in the GEN PSYCH and NON-PSYCH samples, while acceptance of the doctor's competency was stronger in the non-psychiatric subsample. In other words, our findings suggest that psychiatrists have to deal with more intense mistrust which can be partly explained by the assessed population (psychiatric patients hospitalized mainly due to acute decompensation) and this does not correlate with the level of patients' confidence in medication.

Many aspects still need to be elucidated regarding the attitudes of the patients toward medication and healthcare professionals. Clinicians generally tend to overestimate the level of adherence to treatment of patients. More attention needs to be paid to the changeable variables in the background of beliefs and attitudes toward treatment in order to implement efficient adherence-improving interventions. Our results indicate that mental health professionals would need reliable methods for the systematic assessment and modification of patients' attitude toward treatment.

## Data Availability Statement

The datasets generated for this study will not be made publicly available. There is no such an option by the ethical permission.

## Ethics Statement

The studies involving human participants were reviewed and approved by Hungarian Central Ethics Committee. The patients/participants provided their written informed consent to participate in this study.

## Author Contributions

LP recruited the patients, collected data, and prepared the manuscript. JL contributed to the study design, statistical analysis, and review of the manuscript.

## Conflict of Interest

The authors declare that the research was conducted in the absence of any commercial or financial relationships that could be construed as a potential conflict of interest.
